# PLGA-based biodegradable microspheres in drug delivery: recent advances in research and application

**DOI:** 10.1080/10717544.2021.1938756

**Published:** 2021-06-29

**Authors:** Yue Su, Bolun Zhang, Ruowei Sun, Wenfang Liu, Qubo Zhu, Xun Zhang, Rongrong Wang, Chuanpin Chen

**Affiliations:** a Xiangya School of Pharmaceutical Sciences, Central South University, Changsha, China; b Hunan Zaochen Nanorobot Co., Ltd, Liuyang, China; c Hunan Institute for Drug Control, Changsha, China

**Keywords:** Biodegradable microspheres, PLGA, preparation methods, drug delivery, applications

## Abstract

Biodegradable microspheres have been widely used in the field of medicine due to their ability to deliver drug molecules of various properties through multiple pathways and their advantages of low dose and low side effects. Poly (lactic-co-glycolic acid) copolymer (PLGA) is one of the most widely used biodegradable material currently and has good biocompatibility. In application, PLGA with a specific monomer ratio (lactic acid and glycolic acid) can be selected according to the properties of drug molecules and the requirements of the drug release rate. PLGA-based biodegradable microspheres have been studied in the field of drug delivery, including the delivery of various anticancer drugs, protein or peptide drugs, bacterial or viral DNA, etc. This review describes the basic knowledge and current situation of PLGA biodegradable microspheres and discusses the selection of PLGA polymer materials. Then, the preparation methods of PLGA microspheres are introduced, including emulsification, microfluidic technology, electrospray, and spray drying. Finally, this review summarizes the application of PLGA microspheres in drug delivery and the treatment of pulmonary and ocular-related diseases.

## 1. Introduction

The traditional drug delivery system is difficult to achieve accurate control of the drug release rate and release location (Li et al., 2014). After administration, the drug may flow through the whole body along with blood, and the blood drug concentration fluctuates greatly. Generally, the content of the drug in plasma is very high at the initial stage of administration, but it will drop below the effective blood concentration soon, which requires the patient to take drugs for a long time and at high frequency to maintain the effect of drug therapy. Based on the above situation, a new drug delivery system (DDS) is proposed, which can slowly and continuously release the drug or keep the drug at a constant release rate, and finally deliver the drug to the target location in the body (Patil et al., 2018; Zhou et al., 2020). Compared with traditional administration methods, DDS can prolong the therapeutic effect of drugs, reduce the number of medications, improve patient compliance, and reduce the systemic side effects of drugs (Ozeki et al., 2012; Wen et al., 2013; Prajapati et al., 2015; Liang et al., 2020; Malakinezhad et al., 2020; Ren et al., 2021; Tian et al., 2021; Zhang et al., 2021). At present, there are many kinds of new drug delivery systems, microsphere is one of the most common DDS. The microsphere is a kind of particle dispersion system formed by drug adsorption or dispersion in the polymer matrix, ranging in diameter from 1 to 1000 μm (Karan et al., 2020). It has many advantages as DDS because of its rich structure and function. It can be administered through a variety of routes, such as subcutaneous or intratumoral injection, pulmonary delivery as an inhalable agent, etc. Moreover, by encapsulating the active pharmaceutical ingredient (API) in the polymer matrix, the microsphere can prevent the API from being inactivated by environmental (temperature, PH, and oxidative effects) stimuli, as well as mask the unpleasant smell of API (Lengyel et al., 2019). Microspheres can be divided into two types: biodegradable and non-biodegradable according to different carrier materials. Non-biodegradable microspheres will accumulate in the body after administration and cause inevitable toxicity (Prajapati et al., 2019), while the biodegradable microspheres can automatically degrade into products that are nontoxic to the human body, without the need for a second operation to remove the residual polymer matrix in the body (Li et al., 2018). At present, there are many studies on the use of biodegradable microspheres for continuous or targeted drug delivery, involving a wide range of drug types, including anticancer drugs (Ozeki et al., 2012; Alange et al., 2017; Ni et al., 2020), proteins or peptides (Zhai et al., 2015; Yang et al., 2020; Zhou et al., 2020), and plasmid DNA (Herrmann et al., 2015; Terry et al., 2019; Lu et al., 2020). Biodegradable microspheres have become an integral part of new drug delivery systems.

The key to the composition of biodegradable microspheres lies in the application of biodegradable polymer materials. The most important property of such materials is their biodegradability. The substances produced by human metabolism will neither cause harm to the human body nor harm to the environment (Prajapati et al., 2019). There are many types of biodegradable polymer materials, including those from natural sources and synthetic ones. In recent years, more and more synthetic biodegradable polymers have been used as carriers for therapeutic drug delivery devices, especially Poly (lactic acid-co-glycolic acid) (PLGA), due to its biodegradability and good biocompatibility, as well as suitable biodegradation kinetics and easy-to-process mechanical properties, have been used as biomaterials since the 1970s, with a long history (Lu et al., 2009). At present, more than 20 kinds of PLGA-based biodegradable microspheres have been approved for use on the market, and many of which are in the stage of research and development or clinical trials. In this development process, many new technologies and achievements have emerged. Therefore, it is necessary to summarize and update the research status and application of PLGA-based biodegradable microspheres, which is beneficial to those researchers and clinicians who are interested in biodegradable microspheres as DDS.

**Figure 1.**
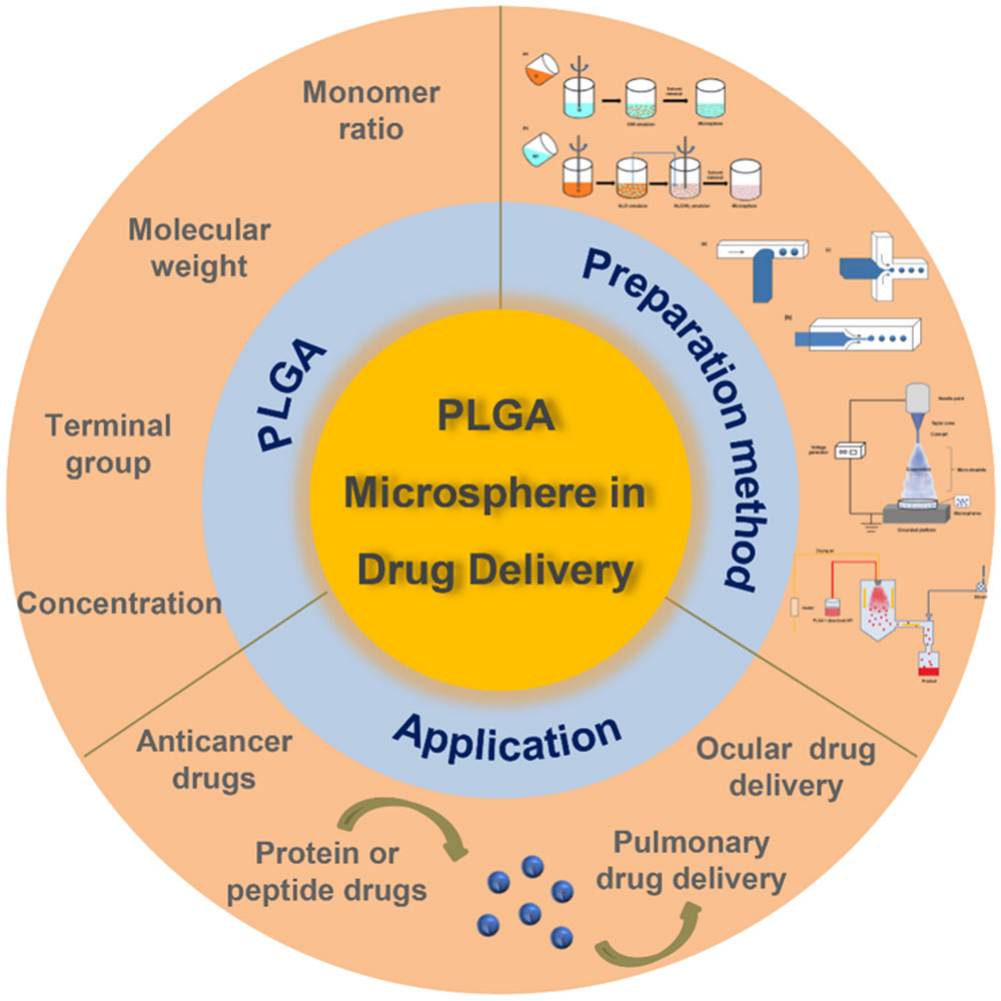
A diagram of PLGA-based biodegradable microspheres in drug delivery.

In this review, we introduce the research on PLGA-based biodegradable microspheres in recent years, focusing on the application of PLGA biodegradable microspheres in cell culture and animal models (Figure 1). Firstly, we discuss the selection of PLGA polymer materials. Then, we introduce the preparation methods of microspheres and summarize the applicability of each method. Finally, we review the application of PLGA biodegradable microspheres in the delivery of anti-cancer drugs and protein or peptide drugs, as well as the treatment of lung and eye-related diseases. Moreover, the encapsulation strategies of different drug delivery are also summarized.

## 2. PLGA as biodegradable microsphere carrier material

There are many kinds of carrier polymers for biodegradable microspheres, including both natural biodegradable polymers and synthetic ones. Most of the natural polymers come from plants, animals or microorganisms, such as starch (Lin et al., 2013; Sommer et al., 2018; Tesfay et al., 2020), chitosan (Li et al., 2017; Calzoni et al., 2019; Huang et al., 2019; Li et al., 2020; Cirri et al., 2021), alginate (Campana et al., 2020; Ghumman et al., 2020; Panchal & Vasava, 2020), gelatin (Kim et al., 2018; Chen et al., 2019; Javanbakht et al., 2019), etc. They are used as microsphere carriers due to their rich properties and good biocompatibility (Prajapati et al., 2015). However, with the development of science and technology, as well as the increasing requirements for the performance of carrier polymers in drug delivery systems, many synthetic biodegradable polymers have been developed. The advantage of synthetic biodegradable polymers lies in that they can adjust the degradation kinetics and mechanical properties of the polymers in the process of synthesis according to the needs of practical applications, which is helpful to develop the diversity of their applications. Compared with natural polymers, the properties and functions of synthetic biodegradable polymers are more predictable (Panchal & Vasava, 2020). PLGA, as a synthetic polymer, is one of the most widely used drug delivery carrier (Zeng et al., 2013; Cheng et al., 2018; Shi et al., 2020), which can transport proteins (Xu et al., 2020; Zhai et al., 2020), peptides (Cai et al., 2019; Zhang et al., 2020; Jin et al., 2021), bacterial or viral DNA (Koerner et al., 2019; Lu et al., 2020), and various anticancer drugs (Lee et al., 2018; Shakeri et al., 2020). PLGA is synthesized by random polymerization of lactic acid (LA) and glycolic acid (GA), which has good biocompatibility and biodegradability (Peng et al., 2018; Ma et al., 2020). PLGA is hydrolyzed into lactic acid and glycolic acid in vivo, and further metabolized into water and carbon dioxide through the tricarboxylic acid cycle, and finally excreted in the lung. The safety of PLGA has been recognized by the United States Food and Drug Administration(FDA) and the European Medicines Agency, and has now been officially included by the FDA as a pharmaceutical excipient (Han et al., 2016; Ruman et al., 2020; Stefani et al., 2020).

The properties of PLGA can be changed by adjusting the monomer ratio (LA/GA), molecular weight (M_W_), concentration, and terminal group, which will affect the encapsulation efficiency (EE%) and drug release kinetics of PLGA microspheres (Li et al., 2021). The polymer PLGA retains some properties of the two monomers, such as the rigidity, hydrophobicity, and slow degradation of LA and the extensibility, relatively less hydrophobicity, and faster degradation of GA (Essa et al., 2020). Generally speaking, increasing the content of the GA monomer in PLGA polymer can improve the hydrophilicity of the polymer, and the amorphous degree of the polymer structure will also increase correspondingly (Kim et al., 2019; Koerner et al., 2019; Essa et al., 2020). However, there are some special cases. Studies have shown that when the ratio of two monomers is 50:50, the polymer degrades at the fastest rate. This is because PLGA50:50 has the lowest crystallinity and high hydrophilicity, so it is helpful for water to penetrate the polymer matrix (Hsu et al., 2019; Koerner et al., 2019; Essa et al., 2020). PLGA polymers with high molecular weight have a faster degradation rate due to their better structural integrity (Wan & Yang, 2016; Essa et al., 2020). Besides, increasing the concentration of PLGA will increase the particle size of microspheres (Han et al., 2016). For PLGA terminated by different functional groups, its hydrophilicity and degradation rate will also be different. Generally speaking, polymers containing free -COOH groups are more hydrophilic (Wan & Yang, 2016), and ester-terminated PLGA has better hydrolysis resistance than acid-terminated PLGA, and the degradation cycle becomes longer (Kim et al., 2019; Wang et al., 2019). In this review, the differences in particle size, encapsulation rate, and release kinetics of microspheres prepared by PLGA with different properties were listed, as shown in Table 1. In addition to regulating the PLGA itself, the PLGA surface can be modified to change its hydrophilicity or increase its targeting capability (Li et al., 2020). For example, the PLGA surface can be modified to target ligands, such as antibodies or small molecular substances, or to connect some reaction units (amine, viny, and maleimide, etc.) to the end of the PLGA copolymer (Kim et al., 2019).

**Table 1.** Effects of PLGA composition on particle size, drug loading, and release profile of microspheres.

Encapsulated drug	PLGA	Particle size	EE%	Release profile	References
Ciprofloxacin HCl	Different PLGA feeding amount in 5 ml of Acetonitrile, viz 100, 200, 400 mg	42.3, 59.7, and 62.2 μm respectively	EE% of 90.0, 93.8, and 95.3% respectively, positive correlation with PLGA concentration	The drug release rate was decreased	(Jeong et al., 2008)
Etanidazole	The concentration of PLGA in the organic solvent (Ethylacetate or DCM) from 1% to 7% (w/v)	Ethyl acetate(EA): hardly varies with the polymer concentration, except for the polymer PLGA 50:50 DCM: particle size increased with increasing polymer concentration	EE% generally decreases with increasing polymer concentration EA can achieve EE% higher than DCM	Increasing polymer concentration can prolong the release duration DCM always achieves a faster release rate than EA	(Wang & Wang, 2003)
	In the order of PLGA 50:50, PLGA 65:35, and PLGA 85:15	The particle size decreased	–	The release rate of microspheres decreased	
Ganciclovir(GCV) and its lipophilic prodrug (GCVMB)	In the order of PLGA 50:50 and PLGA 65:35 (M_W_: 45,000–75,000 Da)	GCV: 75.9 ± 1.6 and 85.8 ± 1.5 μm respectively. GCVMB: 65.9 ± 1.0 and 69.7 ± 1.1 μm respectively	GCV: EE% of 88.7 ± 0.86 and 91.3 ± 0.54% respectively GCVMB: EE% of 81.7 ± 0.25 and 85.5 ± 0.67% respectively	Both: PLGA 65:35 degrade at a slower rate as compared to PLGA 50:50	(Janoria & Mitra, 2007)
Doxorubicin (Dox)	In the order of PLGA 50:50 and PLGA 75:25	363.1 and 361.4 nm respectively	EE% of 48.37 and 38.65% respectively	The released drug from PLGA 50:50 NPs and PLGA 75:25 NPs were 70.98 and 62.22 % of the entrapped released drug in 20 days of study	(Amjadi et al., 2012)
Vincristine sulfate (VCR) and Quercetin (QC)	PLGA 50:50 (M_W_: 15 KDa and 17 KDa), PLGA 70:30 (M_W_: 15 KDa, PLGA 75:25 (M_W_: 15 KDa and 17 KDa) The concentration of PLGA in an acetone dichloromethane mixture from 10 to100 (mg/ml)	VCR and QC both have a positive correlation with the molecular weight of PLGA and PLGA concentration.	VCR and QC both have a positive correlation with the molecular weight, concentration, and ratio of PLGA	–	(Song et al., 2008)
Doxycycline Hyclate	PLGA 50:50 with different end group, i.e., Purasorb®PDLG 5002,/5002 A (M_W_: 17 KDa), and 5004/5004 A(M_W_: 44 KDa)	The average size of around 1 µm, regardless of the end group and molecular weight of PLGA	The microspheres made from PLGA with a high molecular weight and ester end group showed a significantly higher encapsulation efficiency	A drug release within 42 days of approximately 92%, 89%, 47%, and 43% for PDLG 5002 A, 5004 A, 5002, and 5004, respectively	(Wang et al., 2019)

PDLG 5002/5004 with the ester end group; PDLG 5002 A/5004A with the acid end group.

As shown in Table 1, not all studies show the same regular changes, some even show opposite trends. This is because the particle size, entrapment efficiency, and release curve of microspheres are not only dependent on the polymer carrier but also related to the physical and chemical properties of the encapsulated drug, the solvent for dissolving the organic phase carrier, and the preparation method (Koerner et al., 2019). Generally, hydrophilic compounds are more difficult to load than lipophilic compounds, and the release rate of hydrophilic substances is faster because lipophilic substances will hinder water molecules from entering the microspheres, thus reducing the degradation rate of polymers. Therefore, when we choose PLGA as the carrier material, we should consider other aspects in addition to the properties of PLGA itself (Janoria & Mitra, 2007; Han et al., 2016).

## 3. Preparation method of PLGA microsphere

### 3.1. Emulsification

Emulsification is the most commonly used method for the preparation of microspheres. It is very friendly to the encapsulation of temperature-sensitive drugs and the operation process is simple, and the particle size can be controlled to a certain degree (Han et al., 2016). The emulsification method can be divided into a single emulsification method (O/W) and double emulsification methods (W_1_/O/W_2_, S/O/W, and W/O/O). According to the different physical and chemical properties of the encapsulated drug molecules, different preparation methods can be selected.

#### 3.1.1. Single emulsification (O/W)

**Figure 2.**
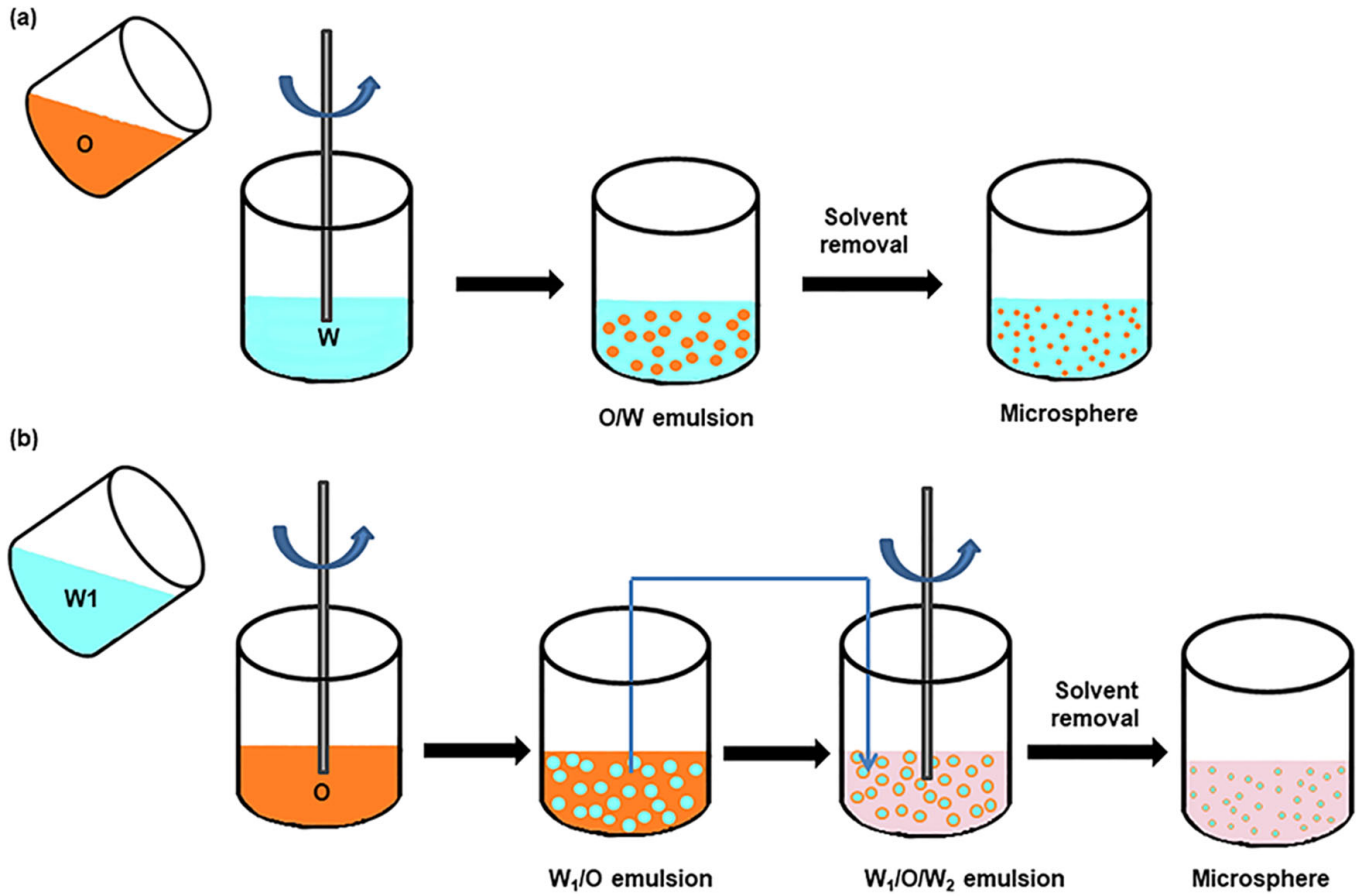
Schematic of PLGA microspheres prepared via (a) single emulsion and (b) double emulsion.

The single emulsification method is usually suitable for hydrophobic drug molecules (Dang & Guan, 2020) (Figure 2(a)). The polymer carrier and drug are dissolved in volatile organic solvents, and then the mixed organic phase solution is added into the aqueous phase containing a surfactant to emulsify under the action of mechanical force. With the volatilization or extraction of organic solvent, the droplet shrinks and solidifies into polymer particles (Ramazani et al., 2016). To achieve higher drug loading and encapsulation efficiency, some water-soluble cosolvents, such as acetone, are usually added into organic solvents (Bee et al., 2018). Although the single emulsification method is simple and easy to operate, the microspheres prepared by this method usually have the problems of wide particle size distribution and poor entrapment efficiency (Zhang et al., 2020).

#### 3.1.2. Double emulsification

Double emulsification is often a strategy used to encapsulate hydrophilic drug molecules (Bee et al., 2018; Lee & Pokorski, 2018). The drug molecule aqueous solution (inner water phase W_1_) is first emulsified with the polymer organic phase solution to form W_1_/O droplets, and then it was added into external water phase W_2_ containing a surfactant to further emulsify to form W_1_/O/W_2_ droplet (Figure 2(b)). As the organic solvent is removed, the droplets solidify into microspheres (Iqbal et al., 2015; Ramazani et al., 2016). However, the prepared drug-loaded microspheres usually have poor encapsulation rates and high initial outbreak rates due to the high water-solubility of the encapsulated drug, which leads to the diffusion of the drug into the external aqueous phase before solidification (Iqbal et al., 2015). Studies have shown that the initially formed W_1_/O droplets are very important to the final droplet stability (Bee et al., 2018), which can be improved by reducing the ratio of V_W1_/V_O_ or adding stabilizers to the internal water phase (Han et al., 2016; Ramazani et al., 2016). Moreover, the stability of double emulsion droplets can also be improved by increasing the lipophilicity of the encapsulated drug or even directly using the solid particles of the drug for encapsulation, that is, the S/O/W emulsification method (Giri et al., 2013). These strategies can reduce the possibility of drug leakage to the external water phase. However, it should be noted that the size of the target drug encapsulated in the S/O/W emulsification method must be micron (Bee et al., 2018). Besides, the droplet can be stabilized by changing the pH of the external water phase. For example, F. Ramazani et al. (Ramazani et al., 2015) increased the pH value of the external aqueous phase from 5 to 9 (greater than the highest pKa of the encapsulated drug imatinib), and the drug loading efficiency of the PLGA microsphere correspondingly increased from 10% to 90%.

In addition to the above measures, there is another strategy to prevent drug molecules from leaking into the outer water phase. In W/O/O emulsification, an oil phase that is immiscible with the polymer organic phase is used instead of the external water phase in the original method, so that the encapsulated water-soluble compounds will not diffuse into the oil-soluble medium (Giri et al., 2013). That is to say, the drug solution and polymer carrier solution are emulsified and then added to another immiscible oil phase, and then the microspheres with high encapsulation efficiency can be obtained.

### 3.2. Microfluidic technology

**Figure 3.**
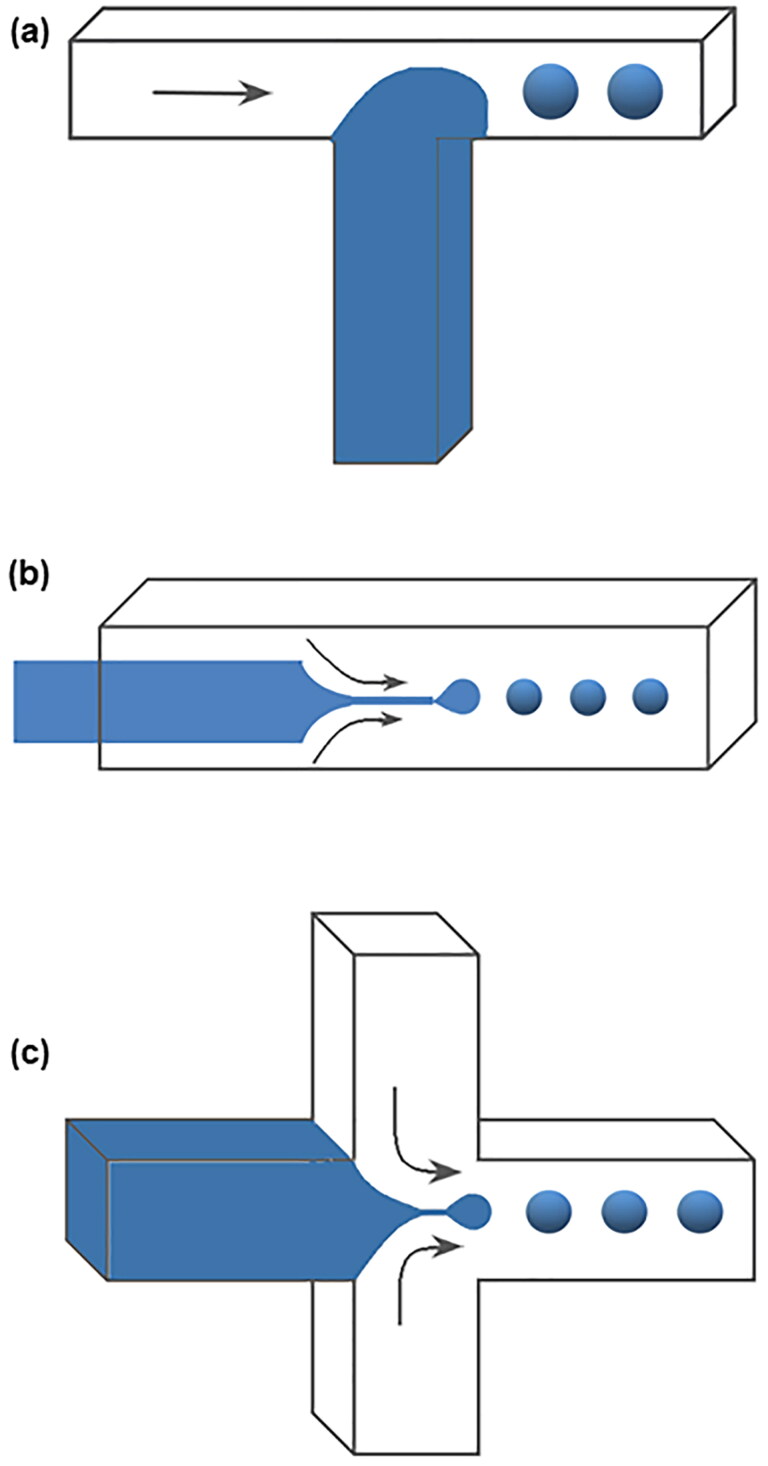
Schematic of microfluidic devices for making PLGA microspheres. (a) T-Junction microfluidic device, (b) co-flow microfluidic device, (c) flow-focusing microfluidic device.

**Figure 4.**
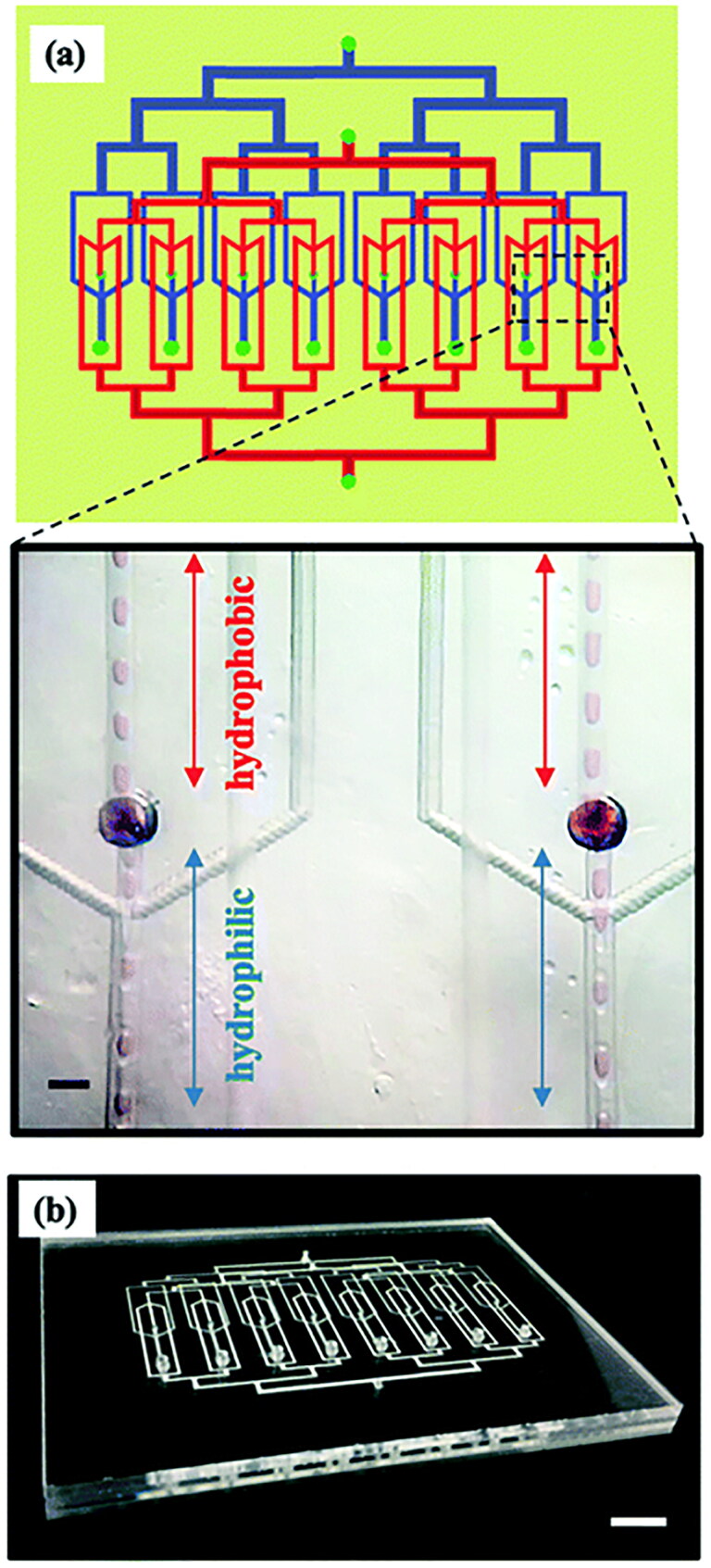
(a) Schematic of a microfluidic parallelization device containing 8 double emulsion drop makers. Hydrophobic channels (indicated in red) and hydrophilic channels (indicated in blue) are located on different layers of the device. Inset shows the simultaneous operation of two adjacent drop makers of the parallel chip. (b) Photograph of the parallelized microfluidic device. Adapted with permission from Reference (Nawar et al., 2020). Copyright 2020, Royal Society of Chemistry.

Microfluidic technology is an emerging strategy for the production of highly controlled and uniform microspheres (Li et al., 2018; Brzeziński et al., 2019; Kim et al., 2019; Zou et al., 2019; He et al., 2020; Peng et al., 2020; Zeng et al., 2020; Caballero Aguilar et al., 2021). The entire process of droplet formation is performed in a microfluidic device containing micron-sized channels. Immiscible continuous phases (such as a drug-polymer solution and a surfactant-containing aqueous phase) are injected at different independent entrances, and the particles can form at the intersection of channels in different phases due to high shear forces (Han et al., 2016; He et al., 2021). The microfluidic devices have channels of different shapes, among which the most commonly used geometrical shapes for the preparation of microspheres are T-shaped channel, co-flow channel, and flow-focusing channel. In the T-Junction geometry, as shown in Figure 3(a), the dispersed phase flows through one channel while the continuous phase flows through another channel. The two channels are perpendicular to each other, and the microdroplet is formed at the junction of the channel (Essa et al., 2020). The device with the microchannel has a simple preparation process and is suitable for the production of microdroplets with high monodispersity (Li et al., 2018). In the co-flow geometry, as shown in Figure 3(b), the dispersed phase and continuous phase flow in the same direction in different channels, and microdroplets are periodically generated in a dripping pattern at the tip of the orifice of the dispersed phase channel (Duncanson et al., 2012). The size of the microdroplets is usually larger than the tip diameter (Li et al., 2018). The flow-focusing geometry is similar to the co-flow system on the whole, but there is a flow-focusing unit in the flow-focusing system. The continuous phase flows from two sides in the direction perpendicular to the dispersion phase, and the microdroplet is formed under the action of shear force (Li et al., 2018; Kim et al., 2019), as shown in Figure 3(c). In the microfluidic system, the chemical composition of the droplets can be changed according to the change of the constituent fluid (Duncanson et al., 2012). The droplet size can also be controlled according to the flow rate of different phases, which makes the technique very suitable for the preparation of highly complex drug delivery carriers (Rezvantalab and Keshavarz Moraveji, 2019; Peng et al., 2020). Also, microfluidic devices with specific shape microchannels can be designed to meet the functional and structural needs of different microspheres to realize the customization of microspheres (Li et al., 2018). However, the microfluidic method used in the experiment often has low production efficiency and can not achieve mass production (Kim et al., 2019; Rezvantalab and Keshavarz Moraveji, 2019; Peng et al., 2020). Therefore, some improvement measures are needed to solve this problem. Saraf Nawar et al. (Nawar et al., 2020) proposed to parallelize the microfluidic device to realize a chip containing multiple droplet generators at the same time, as shown in Figure 4. Such a droplet generator with a multi-layer geometry structure can greatly simplify the droplet production process and improve production efficiency (Peng et al., 2020).

### 3.3. Electrospray

**Figure 5.**
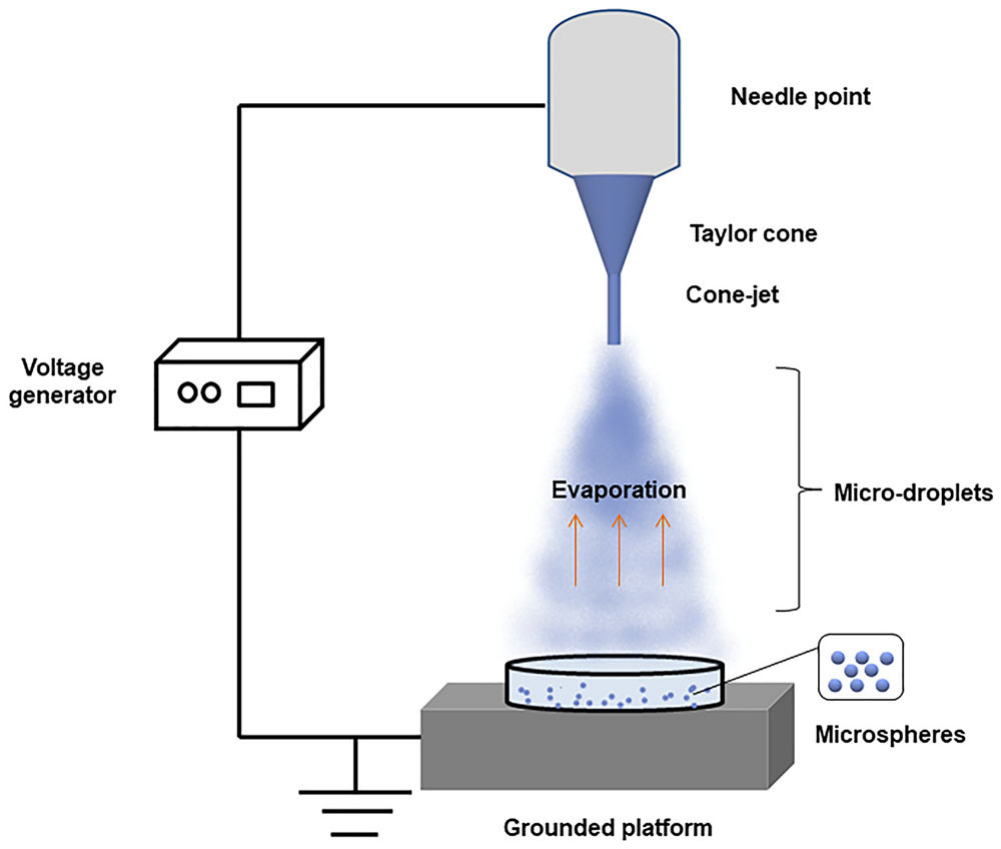
Schematic of the electrospray process for preparing PLGA microspheres.

Electrospray is generally composed of a high-voltage power supply, syringe pump, metal nozzle, and collection substrate (Nguyen et al., 2016; Morais et al., 2020) (Figure 5). The polymer solution is injected into the capillary nozzle by a syringe pump. At this time, a certain voltage is applied between the nozzle and the collecting substrate to generate a high-pressure difference (Jafari-Nodoushan et al., 2015). The ejected solution forms a special ‘Taylor cone’ at the nozzle to form droplets and further atomize into particles, which then move to the collection substrate with opposite charges (Malik et al., 2016; Morais et al., 2020). Generally, the size of the formed particles is between tens of nanometers to hundreds of microns and has a smaller particle size distribution (Nguyen et al., 2016; Morais et al., 2020). Common ES systems include mono-Coaxial ES (MES), coaxial ES (CES), and multiplexed ES (MCES). CES uses an injection pump to transfer the carrier solution (outer layer) and drug solution (inner layer) into the same nozzle from two independent feeding channels. The outer layer solution can wrap the inner layer solution to produce particles with a core-shell structure (Han et al., 2016). The system setup of MCES is composed of several parallel capillary tubes, which is helpful for the improvement of microsphere yield and the realization of large-scale production (Pathak et al., 2016; Morais et al., 2020). Microspheres prepared by electrospray technology can produce particles with different sizes and morphologies by regulating different experimental parameters, such as voltage and velocity (Lai et al., 2017; Wang et al., 2019; Gupta & Panigrahi, 2020). Electrospray technology can achieve one-step preparation of microspheres with high entrapment efficiency (Liu et al., 2020). The whole process is simple and does not cause significant losses, and the encapsulation of sensitive substances such as proteins and cells is also feasible (Han et al., 2016; Nguyen et al., 2016).

### 3.4. Spray drying

**Figure 6.**
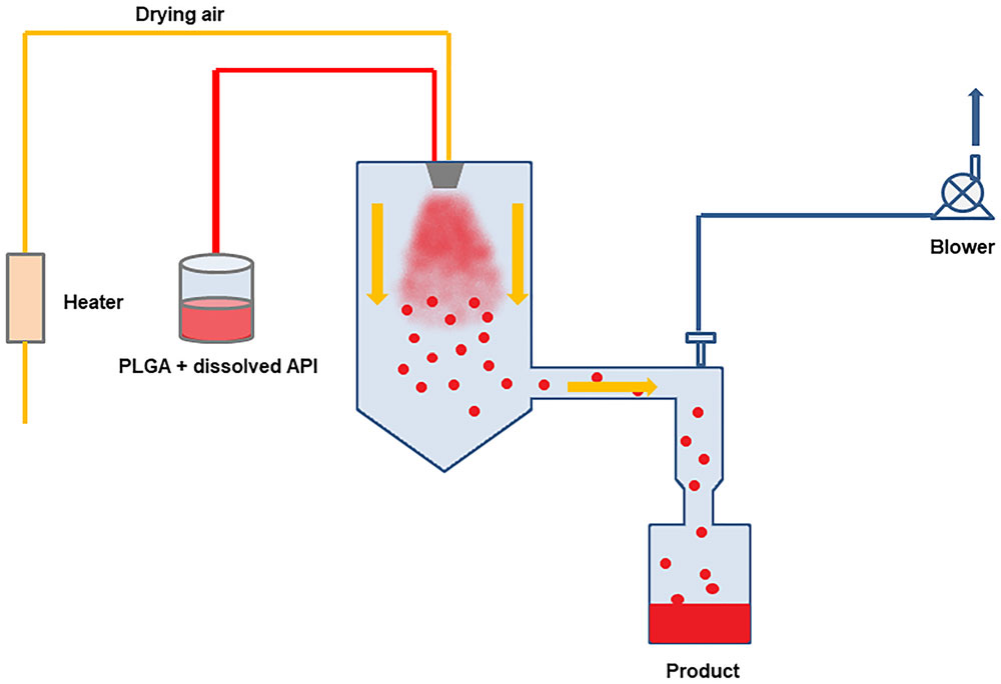
Schematic diagram of the spray drying process.

Spray drying is a technique in which drugs and polymer solutions, suspensions, or emulsions are atomized through a nozzle and injected into hot air (Wei et al., 2020) (Figure 6). As the solvent rapidly evaporates, the atomized droplets are transformed into solid particles (Shi et al., 2020; Wei et al., 2020). The spray drying process usually includes four stages: atomization, droplet mixing with dry gas, solvent volatilization, and product separation, in which the technological conditions and parameters of the atomization process play a decisive role in the droplet size distribution (Ré, 2006; Liu et al., 2015; Deshmukh et al., 2016; Messa et al., 2020), such as the selection of atomizer, nozzle pressure, feed or air flow rate, etc (Bowey & Neufeld, 2010; Shi et al., 2020). The spray drying method can generate particles in one step quickly and continuously, eliminating the need for a separate drying process in other preparation methods (Helbling et al., 2020; Ozturk & Kiyan, 2020; Yurtdas & Ozturk, 2020). The entire preparation process is automated, and a relatively high encapsulation rate can be obtained (Calegari et al., 2020), which is suitable for the encapsulation of proteins or peptides (Aydin, 2020; Shi et al., 2020), plasmid DNA (Walter et al., 2001; Atuah et al., 2003; Jilek et al., 2004), and small molecule drugs (Waldron et al., 2014). Since no external solvent phase is used, it is suitable for most drug molecules whether they are hydrophilic or hydrophobic (Bee et al., 2018).

## 4. Application

PLGA microspheres have broad application prospects in controlling drug release and achieving targeted drug delivery. In this section, we focus on the latest development of PLGA microspheres in anticancer drugs and protein or peptide delivery. To achieve the precise treatment of cancer, drugs are usually required to be delivered to the target site within an appropriate time range to achieve effective tumor treatment and low systemic toxicity. Therefore, it is necessary to build targeted PLGA microspheres delivery system for different disease treatments. As for protein or peptide drugs, due to their physical and chemical properties, they are easily affected by enzyme degradation and often can not be effectively absorbed into the systemic circulation to achieve therapeutic effects. PLGA microspheres can improve the stability of protein or peptide drugs in the internal environment by encapsulation. Strategies to improve the loading efficiency of protein or peptide drugs in microspheres are also discussed. The particularity of the delivery site often has different requirements on the size and surface modification of the microspheres, such as ocular and pulmonary drug delivery. In this section, we also summarize the latest developments in the administration of PLGA microspheres to the eye and pulmonary.

### 4.1. PLGA microsphere in cancer drug delivery

The most common application of PLGA-based biodegradable microspheres is the delivery of drugs for cancer treatment (Table 2). Due to the complexity of cancer pathology, conventional chemotherapy treatments are not effective and have toxic side effects on normal tissues and organs. The use of PLGA microspheres for drug delivery can design corresponding drug-carrying systems according to the characteristics of different cancers, improve the targeting and therapeutic effects of drugs, and extend the time of drug effect.

**Table 2.** PLGA-based biodegradable microspheres for cancer drug delivery.

Drugs	PLGA composition	Preparation method	Sustained drug release	Cell model	Animal model	Cancer	References
Verteporfin	PLGA 50:50 (M_W_: 7-17 KDa) and PLGA 85:15 (M_W_: 190-240 KDa)	O/W emulsification	–	GBM1A, IOMM-LEE, KT21-MG1, JHC7, IOMM LEE , and KT21-MG1 cells	GBM xenograft model (male nude mice)	Intracranial tumor	(Shah et al., 2019)
Paclitaxel	PLGA 75:25 (M_W_: 24483)	W_1_/O/W_2_ emulsification	12 days with a cumulative release of 85%	U251 cells	Human liver carcinoma xenograft model (BALB/c-nu mice)		(Zhang et al., 2018)
Camptothecin/rvincristine	PLGA 75:25 (M_W_: 14400)	W_1_/O/W_2_ Emulsification	**CPT:** 72.5% (28 days) **VCR:** 54.5% (28 days)	–	Rat glioma models		(Ozeki et al., 2012)
Quercetin	PLGA 50:50	O/W emulsification	**PH7.4：**the amount of quercetin release was 67.81% in 12 h **PH5.3：**a cumulative drug release percentage of 72.21% in 12 h	THP-1 cell lines and MCF-7 lines	–	Breast cancer	(Karthick et al., 2019)
Curcumin	PLGA 50:50	O/W emulsification	–	–	A transgenic mouse model of HER-2 cancer		(Grill et al., 2018)
Doxorubicin	PLGA 50:50 (M_W_: 10000)	W_1_/O/W_2_ emulsification	–	The mouse breast cancer cell line 4T1	4T1 tumor model (female BALB/c nude mice)		(Fang et al., 2015)
Doxorubicin hydrochloride	PLGA (M_W_: 54,600 Da)	Coaxial electrostatic spray	A cumulative release amount of over 80% in 21 days	Endothelial cells and 4T1 cells	4T1 tumor model (female BALB/C mice)		(Ni et al., 2020)
Doxorubicin	PLGA 50:50 (RG 503,M_W_: 33,000 Da)	Electrospray	A cumulative release amount of 85.8 % after 30 days	–	Male Sprague Dawley rats	Tumor	(Hsu et al., 2020)
Camptothecin	PLGA (M_W_: 24,000 Da)	W_1_/O/W_2_ emulsification	**—**	HeLa cancer cells	–	Cervical cancer	(Ayyanaar et al., 2019)
7-Eth-10-hydroxy camptothecin	PLGA 50:50 (M_W_: 15,000)	O/W emulsification	**—**	–	H22-bearing KM mice (female Kunming mice)	Tumor	(Hao et al., 2019)
5-fluorouracil	PLGA 50:50 (M_W_: 47 KDa)	S/O/Ho method	One month	–	Male rabbits with colon tumors and adult male Sprague Dawley (SD) rats	Colon tumor	(Lin et al., 2014)

#### 4.1.1. Intracranial tumor therapy

**Figure 7.**
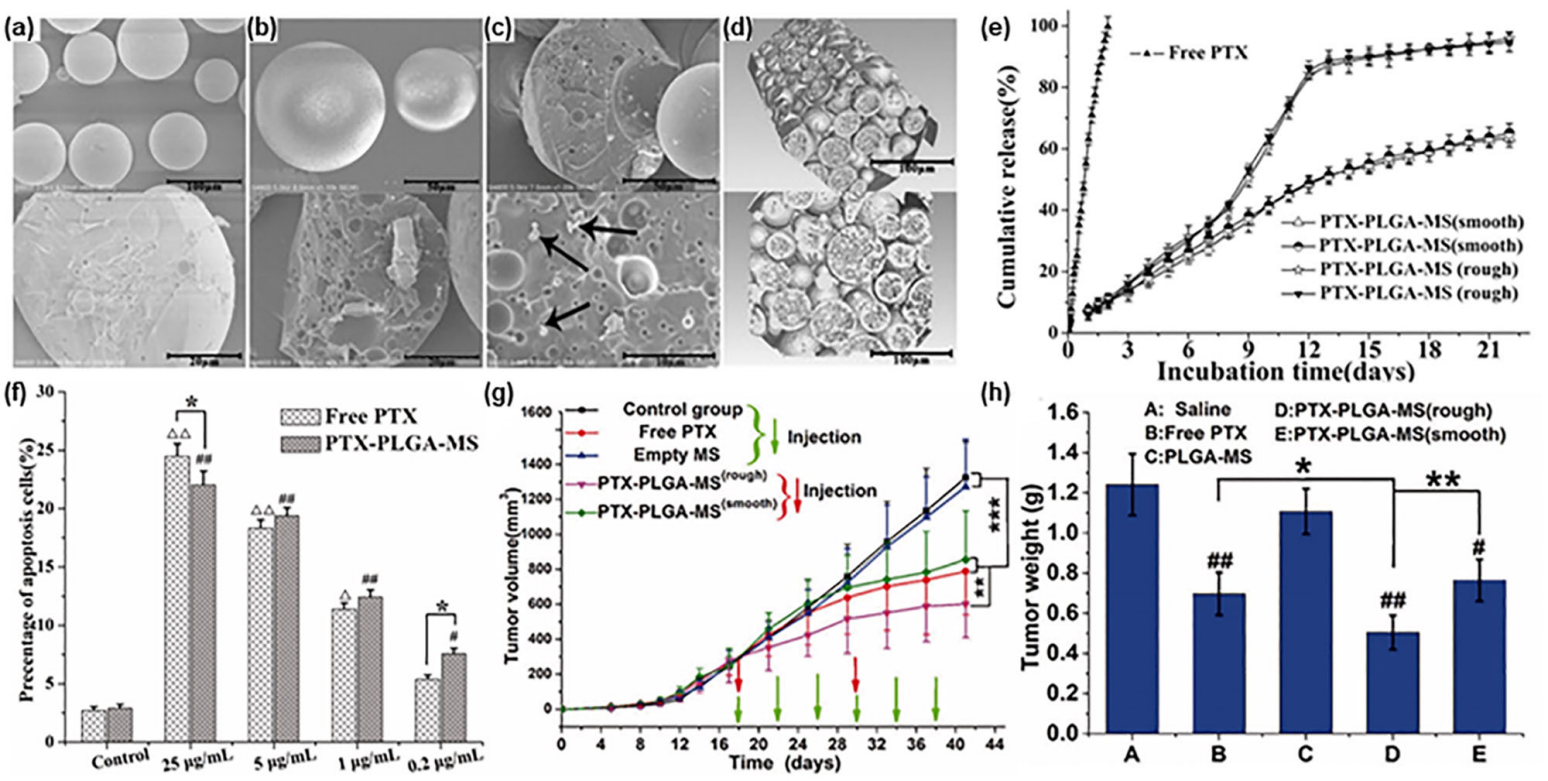
(a) The SEM micrographs of smooth PTX-PLGA-MS. (b-d) The SEM micrographs of rough PTX-PLGA-MS and the arrows represent the PTX drug substances. (e) In-vitro cumulative released curves of free PTX and PTX-PLGA-MS. (f) In-vitro evaluation for apoptosis when PTX formulations co-cultured with U251 cells. (g) Tumor growth curves of smooth or rough PTX-PLGA-MS, PLGA-MS, free PTX, and with no treatment during the entire experiment. (h) Tumor weight of each treatment group at the end of the tests. Adapted with permission from Zhang et al. (2018). Copyright 2018, Informa UK Limited.

The most common and aggressive malignancy in brain tumors is Glioblastoma Multiforme (GBM)( Duwa et al., 2019; Gong et al., 2019; Le Reste et al., 2020; Rahn et al., 2020). Currently, surgical resection, chemotherapy, and radiation therapy are mainly used for treatment, but the tumor often regrowth and the survival rate of GBM patients is low (Jeong et al., 2008; Floyd et al., 2015; Hotchkiss & Sampson, 2021). In drug delivery, drug molecules often encounter the obstruction of the blood-brain barrier, coupled with tumor drug resistance caused by repeated administration, making it difficult to completely remove the tumor (Floyd et al., 2015). By implanting drug-loaded microspheres at the tumor growth/resection site, it can directly bypass the blood-brain barrier and release anti-cancer drugs slowly and long-term. Sagar R. Shah et al. (Shah et al., 2019) embedded verteporfin (VP) in a PLGA carrier to form biodegradable microspheres for the treatment of brain tumors. They reported that VP can increase the radiosensitivity of GBM cells and have anti-proliferative effects on GBM cells. In the in-vitro cell experiment, VP released from PLGA microspheres was still active. They implanted the microspheres into the mice, which had established a tumor model subcutaneously on the right side. Compared with the control group, the VP-PLGA group had significantly less tumor growth. In another study, Zongrui Zhang et al. (Zhang et al., 2018) (Figure 7) prepared paclitaxel (PTX) coated PLGA microspheres (average Particle size of 53.47 ± 2.87 μm, EE%=92.82%) with a porous surface and an internally porous structure. They compared the rough structure microspheres with smooth PTX-PLGA-MS and found that rough PTX-PLGA-MS had higher drug loading, encapsulation efficiency, and a faster drug release rate. In the in-vitro anti-tumor activity experiment, rough PTX-PLGA-MS showed a higher anti-tumor effect than free PTX and smooth PTX-PLGA-MS. Implanting drug-loaded microspheres in mice with liver cancer tumor models showed that the tumor inhibition rate of rough MS was 59.27%, which was 1.35 times and 1.55 times that of free PTX and smooth MS, respectively. Combined with in-vivo biomolecular research and analysis, they reported that the reason for the above results is that the free PTX group exhibits a negative anti-tumor effect due to high drug use frequency resulting in high blood drug concentration in the body; the low drug loading and slow drug release rate of smooth MS resulted in insufficient drug accumulation in the tumor site, and the anti-tumor effect was not ideal; In rough MS group, PTX was accumulated continuously in the tumor site, which could kill tumor cells by promoting cell cycle and expression of apoptosis mRNA and protein. Besides, they reported that the mechanism by which sustained-release PTX could induce cell apoptosis was by inducing microtubule assembly and overexpression of Bax and Cyclin B1 proteins, leading to the destruction of microtubule dynamics, G2/M phase arrest, and thus triggering cell apoptosis.

In the above two studies, the tumors in the animal models were all inoculated into other parts of the rat (such as subcutaneously) to conduct the in-vivo pharmacodynamic study of the microspheres. Although both were injected intratumorally, the tumor site was not located in the brain. The reliability of the evaluation of this animal model for clinical application is not sufficient. Tetsuya Ozeki et al.(Ozeki et al., 2012) prepared PLGA microspheres loaded with camptothecin (CPT) and vincristine (VCR) respectively, and embedded the prepared microspheres in the TGP gel to achieve slow release of the drug at the target site. TGP formed a gel at the tumor injection site to fix the PLGA microspheres and prevent them from spreading in the brain tissue. They evaluated the therapeutic effect by injecting different doses of drug-loaded PLGA microspheres or drug-loaded PLGA/TGP into Rat glioma models. They reported that compared with the control group (untreated rats, 18 days), the CPT/PLGA/TGP formulation prolonged survival time (24 and 26 days; at 10 and 30 μg CPT/rat, respectively). VCR/PLGA preparations prolonged survival (23.5 days), and VCR/PLGA/TGP preparations further increased survival (28, 33, and 33 days at 1, 3, and 10 μg VCR/rat, respectively). In short, the drug-loaded device improves the survival rate of rats with glioma.

#### 4.1.2. Breast cancer therapy

**Figure 8.**
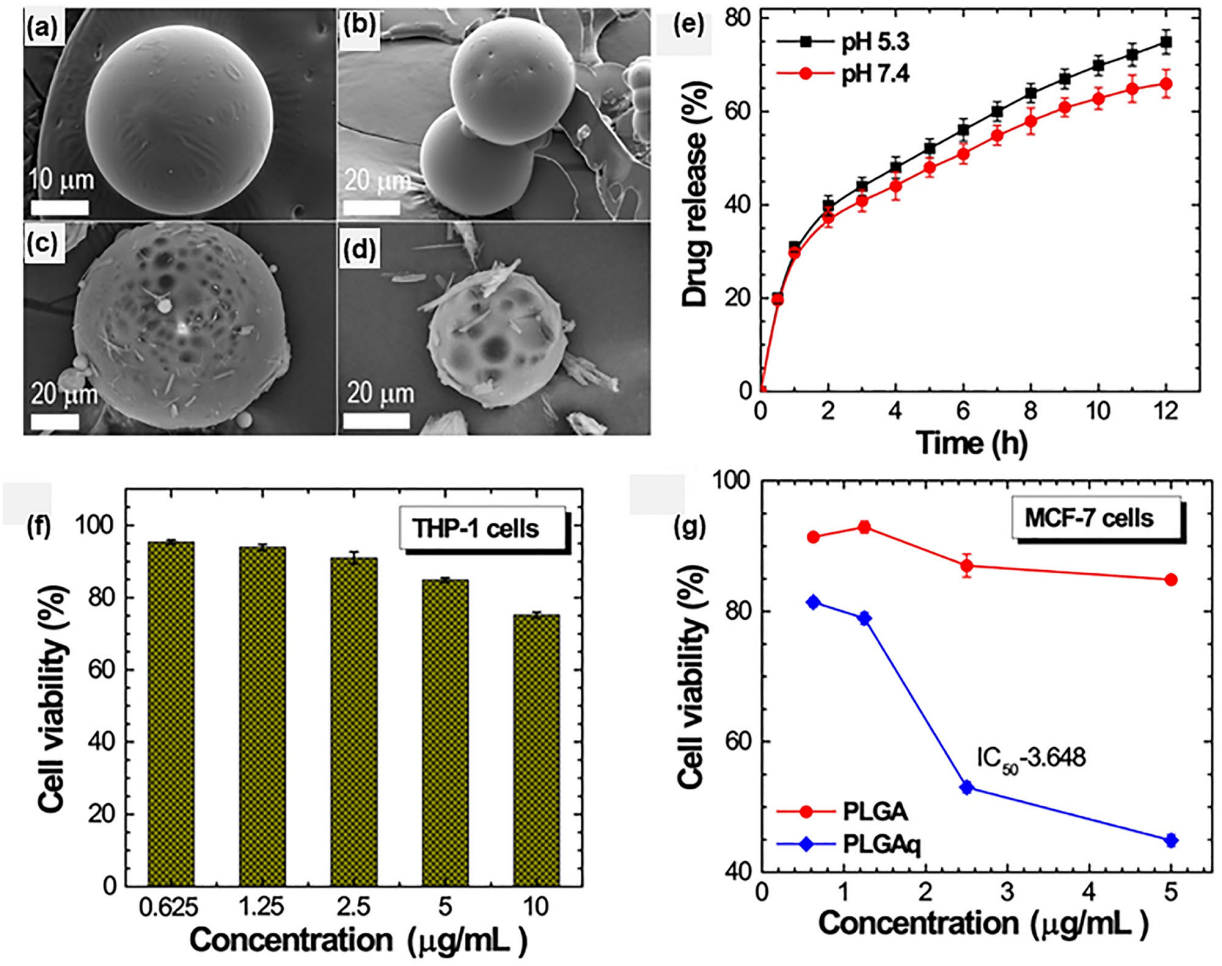
The SEM micrographs of PLGA (a-b) and Quercetin-loaded PLGA (PLGAq) microspheres (c–d). (e) In-vitro cumulative released curves of PLGAq under normal and tumor microenvironment. (f) In-vitro cytotoxicity assay tested using THP-1 cell lines. (g) In-vitro cytotoxicity of PLGA and PLGAq tested on MCF-7 cell lines. Adapted with permission from Karthick et al. (2019). Copyright 2019, Elsevier.

Breast cancer is one of the malignant tumors with the highest incidence in women in the world, and it has seriously threatened women's life and health (Shavi et al., 2017; Bai et al., 2020; Dominguez-de-la-Cruz et al., 2020). At present, there are many studies on the drug delivery system of PLGA microspheres for breast cancer treatment. V. Karthick et al. (Karthick et al., 2019) (Figure 8) isolated Quercetin from onions and loaded it on PLGA microspheres as a drug. The results of the cytotoxicity experiment and flow cytometry detection showed that Quercetin-PLga Microspheres (PLGAq) had an obvious anti-proliferation effect on breast cancer cells. They reported that PLGAq had no toxic and side effects on normal cells(THP-1 cell lines), while in breast cancer cells(MCF-7 lines), PLGAq showed good anti-cancer activity in a concentration range of 0–2 μg/mL, reducing cell activity to less than 40%. In flow cytometry detection, the blank PLGA microspheres with different concentrations (1.5 μg/mL and 3 μg/mL) showed no obvious apoptosis. With the increase of PLGAq concentration, the apoptosis rate of MCF-7 lines also increased, indicating that PLGAq effectively inhibited cell proliferation by inducing apoptosis of breast cancer cells. Alex E. Grill et al. (Grill et al., 2018) tested the anti-breast cancer effect of Curcumin-loaded PLGA microparticles in a transgenic mouse model of HER-2 cancer. They treated mice with drug-loaded microspheres at different stages of tumor growth to determine the best intervention time. They found that compared with the blank microsphere treatment group, mice treated at the second or fourth week of age had a 2–3-week delay in tumor appearance, and a significant reduction in abnormal breast tissue area at 12 weeks of age, demonstrating the anti-cancer effect of curcumin microspheres. However, what was interesting was that they also found that compared with the normal saline treatment group, the blank microsphere treatment group accelerated the formation of tumors, which may be related to the systemic inflammatory response caused by PLGA particles at high doses, and they would further investigated this phenomenon. In another study, a magnetic response drug delivery system based on PLGA microspheres was prepared, which had the effects of chemotherapy and hyperthermia for breast cancer treatment. Kun Fang et al. (Fang et al., 2015) developed magnetic PLGA microspheres coated with doxorubicin (DOX-MMS), where DOX was in the core of the microspheres, and γ-Fe2O3 nanoparticles (IOs) were assembled on the surface of the microspheres by electrostatic deposition. They reported that under an external alternating current magnetic field (ACMF), the IOs of the drug delivery system could generate a thermal effect and increased the permeability of the microsphere shell to release drugs. The cytotoxicity test of breast cancer cells in vitro showed that the survival rates of the DOX-MMS + ACMF (30 min) group and the DOX-MMS group were significantly different (10% and 81.6%, respectively). In the in-vivo experiment, the anti-tumor effect was realized as follows: DOX-MMS + ACMF > MMS + ACMF > DOX-MMS > blank control group, indicating that DOX-MMS combined with hyperthermia can effectively resist breast cancer cells in vivo. Guoli Ni et al. (Ni et al., 2020) also adopted a combination of chemotherapy (Doxorubicin) and thermal therapy. However, different from the previous study, the thermal effect in this study was generated by polydopamine (PDA) nanoparticles embedded in PLGA microspheres under NIR irradiation. They reported that when the microspheres were injected into the body of a mouse with a mammary tumor and irradiated by near-infrared light into the tumor site, PDA nanoparticles would immediately produce local thermal effects, and DOX•HCl loaded in the microspheres could be released rapidly due to the influence of photothermal effect. In in-vivo antitumor experimental results showed that the tumor growth was slowest in the PDA/DOX• Hcl@plga + laser treatment group, the relative average tumor volume was only 1.4 on the 21st day of treatment, and the survival rate of the mice was 100%. An in-vitro cytotoxicity test also showed that the microsphere delivery system did not affect on survival of normal cells.

#### 4.1.3. Other cancers therapy

**Figure 9.**
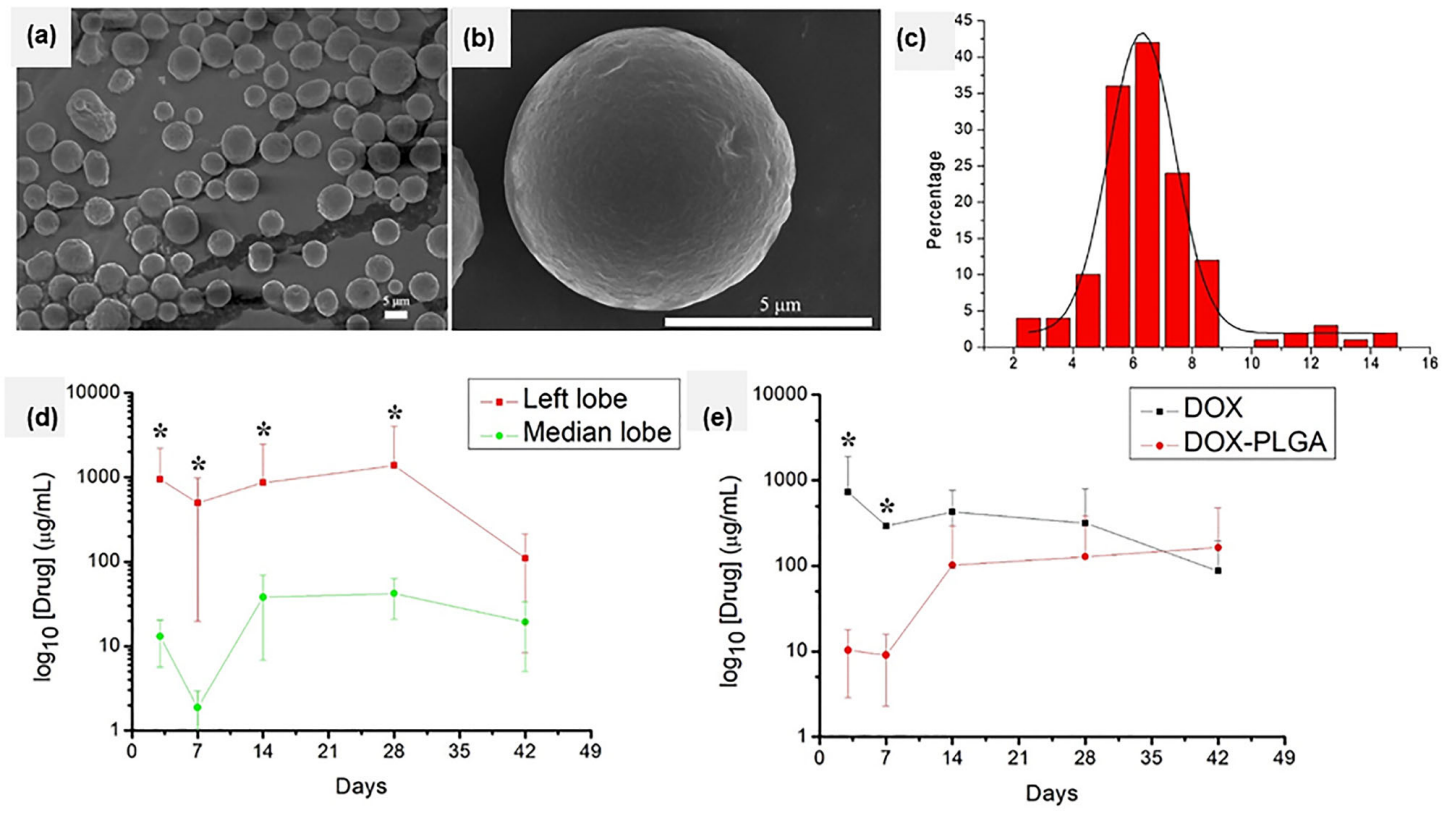
(a, b) The SEM micrographs of microspheres. (c) The size distribution of microspheres. (d) The concentration distribution of DOX in the left and median hepatic lobes of the rats during the experiment. (e) The plasma concentration of DOX in rats after injection of the drug (DOX) – loaded microsphere and free DOX during the experiment. Adapted with permission from Hsu et al. (2020). Copyright 2020, Elsevier.

In addition to brain and breast cancer, some studies have also reported the application of PLGA microspheres in other cancers or malignant tumors. Ming Yi Hsu et al. (Hsu et al., 2020) (Figure 9) prepared doxorubicin-loaded PLGA microspheres (average particle size 6.74 ± 1.01 μ m), which were administered by intratumoral injection for the treatment of unresectable tumors and tumors with an adverse reaction to conventional treatment. They conducted in-vivo pharmacokinetic studies by injecting drug-loaded microspheres into the left lateral lobe of the liver of normal mice and found that the concentration of DOX in the left lateral lobe was always higher than that of the central lobe and peripheral blood, which indicated that it was possible to achieve the local tumor control and the minimization of systemic toxic and side effects. Srinivasan Ayyanaar et al. (Ayyanaar et al., 2019) developed a drug delivery system that released drugs based on the response to reactive oxygen species (ROS) in cancer cells. ROS plays a key role in cell signaling pathways, and oxidative stress caused by metabolic imbalance has been considered a major cause of cancer. In this study, ROS-sensitive tween 80 coated MIONs consisting of anti-cancer drug camptothecin (CPT) were coated with a polymer to form PLGA magnetic microspheres (CPT-MMSs) for the treatment of cervical cancer. They reported that the release rate of CPT in the microspheres in the presence of ROS (H_2_O_2_) was significantly higher than that in the absence of ROS. About 90% of the drug in the ROS group was released after 4.5 hours, while only 40% of the CPT in the absence of the ROS group was released. In the in-vitro cytotoxicity evaluation experiment, the killing effect of CPT-MMSs on human cervical cancer cells (HeLa) was significantly higher than that of the control group (only CPT or tween coated MIONs treated cells), and the results of AO/EB staining assay also showed that HeLa cancer cells treated by CPT-MMSs appeared a large amount of cell apoptosis, and most of the cancer cells treated by the control group were still healthy living cells. In another study, Yanyun Hao et al. (Hao et al., 2019) prepared 7-Eth-10-hydroxy camptothecin (SN-38)-loaded PLGA microspheres for tumor therapy that extended the duration of SN-38 action in the tumor and reduced its concentration in the blood, thereby reducing systemic toxic effects. In the in-vivo pharmacokinetic experiments, they reported that drug-loaded microspheres (SN-38-PLGA-MS) and SN-38 solution (SN-38-SOL) were injected intratumorally into mice respectively. The results showed that the mean residence time (MRT) and Mean Residence Time (CL) values of the SN-38-PLGA-MS group were 4.60 and 0.22 times higher than those of the SN-38-SOL group, respectively, and the peak concentration (Cmax) of SN-38-SOL group in peripheral blood was 3.07 times of that of the SN-38-PLGA-MS group, indicating that microspheres can help prolong the retention time of the drug in tumor and reduce drug leakage. In the in-vivo pharmacodynamics studies, the tumor growth inhibition, tumor-specific growth rate, and tumor doubling time in the SN-38-PLGA-MS group were 1.42, 0.51, and 1.97 times higher than those in the SN-38-SOL group, respectively. Histopathological analysis of tissue also found that H&E sections of tumor tissues of mice showed that SN-38-PLgA-MS had obvious anti-tumor effects and almost no toxic side effects on main organs. QInG Lin et al. (Lin et al., 2014) used a solid-in-oil-in-hydrophilic oil (S/O/hO) novel method to prepare 5-fluorouracil (5-FU)-encapsulated PLGA microspheres for local tumor treatment. The encapsulation rate was >90%, which was significantly better than the W/O/W preparation method. The AUC of 5-FU microspheres was 12 times higher than that of subcutaneous injection. The drug-loaded microspheres were administered to rabbits with colon tumors by intratumor injection and the in-vivo pharmacodynamic study was carried out. The results showed that the inhibition rate of the 5-FU microspheres drug delivery system on tumor volume and tumor growth was better than that of the 5-FU solution group. All of these studies expand the application of PLGA microspheres in cancer treatment and indicate that PLGA microspheres have broad application prospects in the delivery of anti-cancer drugs.

### 4.2. PLGA microsphere in protein or peptide drug delivery

Protein or peptide drugs as therapeutic agents have been widely used in the treatment, diagnosis, and prevention of diseases (Cao et al., 2019), but the physicochemical properties of these drugs are usually unstable, with a short half-life and low bioavailability (Ibraheem et al., 2014; Asfour, 2021), and they are prone to hydrolytic inactivation in complex in-vivo and in-vitro environments (Ma, 2014; Ye & Venkatraman, 2019). Encapsulation of proteins or peptides in the microsphere delivery system can improve the stability and prolong the drug action time to a certain extent. At present, some PLGA microsphere delivery systems that encapsulate such drugs have been approved for clinical application, but microsphere systems encapsulating protein or peptide drugs often have problems such as high initial burst rate and low encapsulation rate, which are related to the physical and chemical properties of protein or peptide drugs. Many strategies have been applied to microsphere delivery systems encapsulating protein or peptide drugs to improve their stability. Heejun Park et al. (Park et al., 2019) improved the stability of PLGA microspheres loaded with exenatide by adding stabilizers, and studied the effects of different stabilizers on the encapsulation rate and release of the microspheres. They prepared microspheres by the W_1/_O/W_2_ solvent evaporation method and compared the stability effects of several different stabilizers at different stages of preparation. They reported that poloxamer 188 was the most effective in exenatide's aqueous solution, contributing to the structural stability of the peptide; phenylalanine can significantly reduce the instability induced by the W/O interface by reducing the adsorption of peptide molecules at the DCM/water interface during W/O emulsification; sucrose could significantly prevent the peptide instability of microspheres during freeze-thaw process. Sucrose and lysine could significantly improve the lyophilization stability of exenatide during freeze-drying; poloxamer 118, lysine, and proline could effectively prevent exenatide from absorbing PLGA. Besides, the pH value could be adjusted appropriately to reduce adsorption capacity. Based on the experimental data in the stability study, they chose the appropriate additive combination formula to significantly improve the encapsulation rate and initial outbreak rate of microspheres. Some studies have shown that it can also be improved by modifying the encapsulated protein or peptide compounds. For example, Jiwei Liu et al. (Liu et al., 2019) reduced the high water solubility of Octreotide acetate by combining Octreotide acetate with dextran sulfate sodium (DSS) to form the hydrophobic ion-pairing (HIP) complex and then embedded the complex in PLGA microspheres by specific solid-in-oil-in-water (S/O/W) method. Meanwhile, diethyl phthalate (DEP) was added to enable the microsphere to repair pores by itself, and a microsphere delivery system with a high encapsulation rate and low initial burst rate was prepared. They reported that compared with the traditional W_1_/O/W_2_ preparation method, the S/O/W method significantly improved the encapsulation rate after the formation of the HIP complex (from 44% to 90%). Under the optimal self-healing conditions, the initial outbreak rate of microspheres prepared by the two methods decreased significantly (W_1_/O/W_2_ and S/O/W decreased from 32.99% to 21.03% and 16.81% to 3.56%, respectively). They also examined the actual drug release behavior of the microspheres in rats, which further confirmed that the self-healing microspheres could not only highly inhibit the initial outbreak, but also had a good drug release effect. Moreover, some studies have been done to improve the stability of the microsphere delivery system loaded with proteins or peptides by modifying carrier polymers. Peng Zhai et al. (Zhai et al., 2015) used PLGA polymer and a hydrophilic biomaterial sodium alginate as a carrier for the delivery of hydrophilic protein drugs. They prepared PLGA/sodium alginate microspheres loaded with bovine serum albumin (BSA) or rabbit anti-laminin antibody protein, and added Span80 during the preparation process to improve stability. They reported that the initial burst release of 5% PLGA/5% sodium alginate decreased by 27% compared with the microspheres containing only 5%PLGA, while there was no significant difference between 5% PLGA/5% sodium alginate and 5% PLGA/3% sodium alginate. The encapsulation rate of microspheres added with sodium alginate was also significantly improved, the encapsulation efficiency of 5% PLGA, 5% PLGA/5% sodium alginate, and 5% PLGA/3% sodium alginate microspheres was 63.79, 74.43, and 75.65%, respectively. They detected the activity of rabbit anti-laminin antibody-protein released from PLGA microspheres and PLGA/sodium alginate microspheres, and found that the activity of proteins released from PLGA microspheres and PLGA/sodium alginate microspheres remained unchanged and had no significant difference, indicating that the addition of stabilizer Span80 in the preparation process was conducive to the preservation of protein activity. They investigated the biocompatibility of the microspheres by using different cell lines. The results showed that PLGA microspheres did not affect cell survival, while PLGA/sodium alginate microspheres had poor biocompatibility with rsc96, 3T3, and L8 cells, which was mainly related to the free calcium ions in the microspheres. Some cells such as ATDC5 cell lines showed a high degree of tolerance to calcium ions. In addition to the modification of carrier polymers and protein or peptide drugs and the addition of stabilizers, the stability of the microsphere delivery system for protein or peptide drugs can also be improved by changing the way of preparation methods. Liqing Chen et al. (Chen et al., 2019) successfully prepared Triptorelin acetate-loaded microspheres with a high rate of encapsulation (71.35%) and a very low initial outbreak rate (0.74%) by using a liquid/oil/oil (L/O/O) phase separation method. Because the phase separation method does not require water phase and high-temperature conditions, it is very beneficial for the stability of peptides or proteins and the improvement of encapsulation efficiency. They first prepared a mixture of the drug and PLGA (dissolved in dichloromethane), followed by phase separation by adding silicone oil to the mixture as a non-solvent, and then transferred the dispersed system to n-heptane for microsphere solidification. By optimization of the process parameters, they obtained the optimal preparation conditions: 2.5% PLGA concentration, 2:1.25 (w/w) Silicone oil (non-solvent) to DCM ratio, 37.5:1 (V/W) N-Heptane (solidification solvent) to DCM ratio, and 400 RPM solidification speed. They reported that the microspheres prepared under the optimal preparation conditions were small in size (50–100 μm), and the in-vitro release of the microspheres showed extremely low initial release (0.78%), the drug was released in the third week and the drug release on the 28th day was about 20%. These studies show that PLGA microspheres can be used as a good carrier for protein or peptide drug delivery, and have great application value (Table 3).

**Table 3.** PLGA-based biodegradable microspheres for protein or peptide drug delivery.

Drugs	PLGA	Preparation method	EE%	Initial burst rate	Cell model	Animal model	References
Exenatide	PLGA 50:50 (Resomer 503H)	W_1_/O/W_2_ emulsification	61.6 ± 0.6%	13.3 ± 0.8%	–	–	(Park et al., 2019)
Octreotide acetate	PLGA (Mw: 2.4–3.8 KDa, free carboxylic acid)	S/O/W emulsification	90%	3.56%	–	Sprague-Dawley Male Rats)	(Liu et al., 2019)
BSA or rabbit anti-laminin antibody-protein	PLGA 50:50 (M_W_: 38–54 kDa) and sodium alginate	W_1_/O/W_2_ emulsification	5% PLGA/3% sodium alginate：75.65% 5% PLGA/3% sodium alginate：74.43%	Reduced by 27% compared to without sodium alginate	RSC96、MC3T3-E1 Subclone 4, and L8 cell line	–	(Zhai et al., 2015)
Triptorelin	PLGA 75:25 (M_W_: 12KDa) with the different concentration, such as 2.5, 5, and 10% respectively	liquid/oil/oil (L/O/O) phase separation	71.35%	0.74%	_	_	(Chen et al., 2019)

### 4.3. PLGA microsphere in pulmonary drug delivery

**Figure 10.**
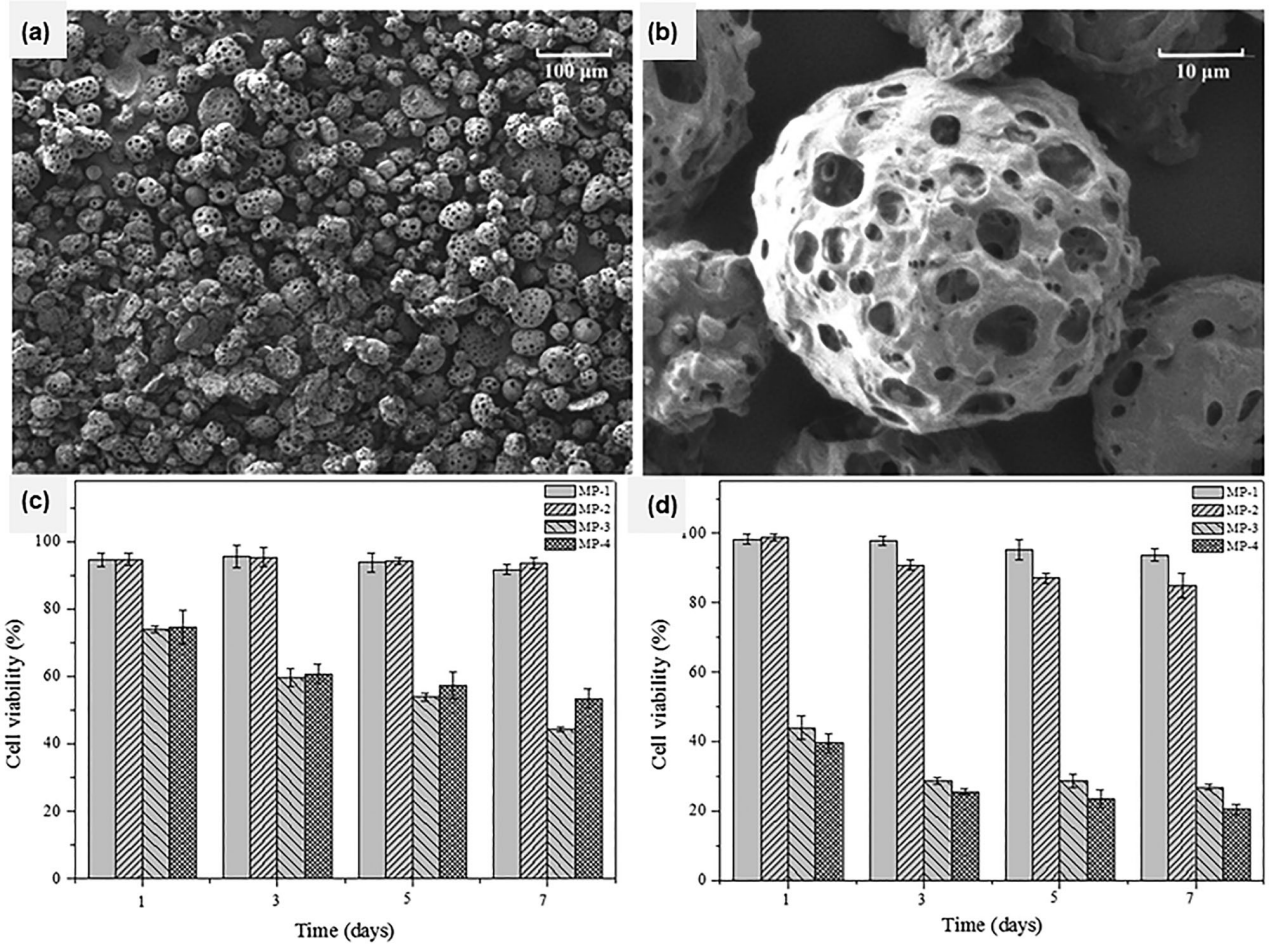
The SEM micrographs of (a) porous PLGA microspheres loaded with doxorubicin and PEI25K/miR-519c (MP-4) and (b) the porous surface, and in-vitro cell viability evaluation of A549 cells treated with the release supernatants from different types of porous PLGA microparticles for 24 h (c) and 48 h (d). Adapted from with permission Wu et al. (2016). Copyright 2016, American Chemical Society.

Drug delivery systems such as microspheres can directly deliver drugs to the lung for the treatment of lung diseases, which can not only improve the drug treatment effect but also reduce systemic toxicity and improve patient compliance (Cook et al., 2005). Transpulmonary administration can avoid the first-pass effect, and due to the large absorption surface area of the lung, thin epithelial cell membrane, and good blood flow supply (Mahajan & Gundare, 2014; Zhao et al., 2017; Chakraborty et al., 2019), it has certain advantages in the treatment of some systemic diseases, such as insulin delivery (Lin et al., 2019). Therefore, the study of transpulmonary drug delivery has a good prospect. Intravenous injection is one of the drug delivery methods for pulmonary targeting microspheres. Generally, microspheres with a diameter equal to or greater than 7 μm can be mechanically filtered through the pulmonary capillary bed and retained in the lung tissue (Wei & Zhao, 2014; Wang et al., 2016). Wenping Wang et al. (Wang et al., 2016) prepared sophoridine (SRI)-loaded PLGA microspheres for the treatment of lung cancer. The average particle size of the microspheres was 17 μm, the encapsulation efficiency was 65%, and about 94% of the drug was released after 14 days of sustained release. They injected SRI-loaded microspheres into the tail vein of rats and then measured the amount of drug distribution in the rat's heart, liver, spleen, lung, kidney, and blood samples at different time intervals. They reported that there were significant differences in tissue distribution of SRI-PLGA microspheres. The drug content in lung tissue was the highest, accounting for 84.1% of the dose. The lung targeting efficiency was 28 times higher than that of the liver, 20 times higher than that of the kidney, and 22 times higher than that of blood. The results showed that the SRI microspheres were capable of lung targeting and slow drug release. In another study, Guozhuan Li et al. (Li et al., 2018) prepared Bisdemethoxycurcumin (BDMC)-loaded PLGA microspheres with a mean particle size of 8.5 μm for targeted therapy of lung cancer. Similarly, they measured the drug content in different tissues at different time points after intravenous injection of drug-loaded microspheres into rats. The results showed that the drug concentration in the lung was higher than that in other tissues and reached the highest concentration at 48 h. In addition to intravenous administration, lung-targeted microspheres can also be administered as inhalable dry powder preparations, which requires the microspheres to have appropriate aerodynamic diameters to ensure that the microspheres are retained in the appropriate positions (Miranda et al., 2018). Marisa C et al. (Gaspar et al., 2019) prepared inhalable levofloxacin (LVX)-loaded microspheres by W_1_/O/W_2_ double emulsion method for the controlled release of LVX in the lung. They investigated the types of PLGA, PVA concentration, and other experimental parameters, and screened out the best experimental conditions. The mass majority diameter (MMAD) prepared with this aerodynamic diameter was 7.1 ± 0.2 μm. Such microspheres could be targeted at specific sites in lung tissues and most of them were able to avoid phagocytosis by pulmonary macrophages. In the in-vitro cytotoxicity test, the survival rate of Calu-3 cells was 70% after being incubated with LVS-loaded MS at the highest detection concentration for 24 h. Considering the possible cytotoxicity of the polymer carrier and the cytotoxicity of LVX itself, the experimental results showed that LVS-loaded MS had low toxicity to normal breathing cells. Most of the obstacles in targeting microspheres as inhalable dry powder preparations to specific sites in lung tissues are due to the complexity of the airway itself, which makes it difficult for the preparation to deposit in the per pulmonary or alveolar areas. Pulmonary macrophages will phagocytose and remove foreign particles deposited in the lung (Gharse & Fiegel, 2016; Li et al., 2019). Therefore, avoidance of clearance by pulmonary macrophages is considered a basic strategy in the design of inhalable preparations (Wu et al., 2016). The porous particles reach the aerodynamic size range by increasing the porosity within the particles, increasing the dispersion of the particles, and achieving efficient deep lung deposition thereby avoiding clearance by pulmonary macrophages (Gharse & Fiegel, 2016). Di Wu et al. (Wu et al., 2016) (Figure 10) prepared porous PLGA microspheres encapsulated doxorubicin and miR-519c with bicarbonate as a porogen. They combined chemotherapy and gene therapy for the treatment of lung cancer. In in-vitro anti-proliferation experiments, they investigated the inhibitory effect of four groups of microspheres on cell proliferation, which were blank porous microspheres group (MP-1), porous PLGA microspheres loaded with PEI25K/miR-519c (MP-2), porous PLGA microspheres loaded with doxorubicin (MP-3), porous PLGA microspheres loaded with doxorubicin and PEI25K/miR-519c (MP-4). They reported that compared with MP-1 and MP-2 groups, MP-3 and MP-4 groups showed a significant anti-tumor effect. The survival rate of cells treated for 48 h with the supernatant of MP-3 and MP-4 groups after release for seven days was only 20%. The survival rate of the MP-3 group was higher than that of the MP-4 group at 24 h. Further studies showed that this was due to the activation of miR-519c, which resulted in the inhibition of ABCG2 expression after 24 h. The concentration of doxorubicin in the MP-4 group was almost twice that in the MP-3 group at 48 h. In addition to porous microparticles, studies have shown that the interaction between PLGA particles and pulmonary macrophages can be regulated by modifying endogenous phospholipids on the surface of PLGA particles. Jiaqi Li et al. (Li et al., 2019) investigated the effects of different types (DPPS, DPPG, and DSPE) and dosage of phospholipids on lung cell uptake, intrapulmonary absorption, and retention behavior of PLGA particles. The researchers used RAW264.7 and NR8383 cells as a macrophage model to study the uptake of lung macrophages in vitro. They reported that PLGA particles modified by pulmonary endogenous phospholipids (DPPS, DPPG, and DSPE) showed an obvious trend of binding with pulmonary macrophages, while pegylated DSPE (PEG2000-DSPE) modified PLGA particles showed obvious poor binding with pulmonary macrophages. Besides, in the study of lung absorption and retention in vivo, when all kinds of phospholipids were at the same molar ratio, they found that DSPE was the best in promoting macrophage phagocytosis. The macrophage uptake of PEG2000-DSPE modified PLGA particles was 209% lower than that of DSPE modified PLGA particles, and the drug content in lung tissue was 4.5 times and 3.5 times higher than that of unmodified particles and DSPE modified particles, respectively. Their study demonstrated that the interaction between PLGA particles and pulmonary macrophages could be regulated and the drug content in the lung could be increased by phospholipid modified microparticles. In addition to being an obstacle to drug delivery under certain circumstances, pulmonary macrophages, as immune system cells that maintain a stable pulmonary environment, are also involved in the pathogenesis of certain respiratory diseases, such as asthma (Yang et al., 2012), pulmonary tuberculosis (Gaspar et al., 2017), and pneumonia(Mehta et al., 2019). Therefore, contrary to the above situation, the treatment of these diseases can be achieved by promoting the combination of PLGA microspheres and pulmonary macrophages. Marcianes P et al. (Marcianes et al., 2019) used labrafil to modify the surface of the prepared PLGA particles and actively targeted Gatifloxacin to macrophages to treat tuberculosis. The prepared drug-loaded PLGA particles had a particle size of 3-5 μm, which was sufficient to deliver the drug to the alveolar level and be taken up by macrophages. They investigated the effects of two different PLGA (PLGA 502 and PLGA 502H) on the preparation of microspheres, and found that PLGA 502H was more favorable for the encapsulation of hydrophilic drugs, and the encapsulation efficiency was up to 89.6%. In the study of the phagocytic behavior of macrophages, they reported that particles modified with or without surface modifiers could be ingested after incubation with cell models for 24 h, but those modified with surface modifiers could be phagocytized in a short time. After 48 hours of co-incubation, the macrophages were still alive and particles could be observed in the cells. Overall, these studies demonstrate promise for the future applications of PLGA microspheres in pulmonary drug delivery (Table 4).

**Table 4.** PLGA-based biodegradable microspheres for pulmonary drug delivery.

Drugs	PLGA composition	Preparation method	Particle size	Sustained drug release	Cell model	Animal model	References
Sophoridine	PLGA 50:50 (Mw: 20KDa)	O/O emulsification	17 μm	14 days	_	SD rats of male	(Wang et al., 2016)
Bisdemethoxycurcum	PLGA 50:50 (Mw: 50000)	O/W emulsification	8.5 μm	96 h	_	Sprague-Dawley rats	(Li et al., 2018)
Levofloxacin	RG 502H (PLGA 50:50, M_W_: 7000–17000, acid termi- nated) and RG 503 (PLGA 50:50, M_W_: 24,000–38,000, ethyl ester terminated)	W_1_/O/W_2_ emulsification	5 μm (MMAD of 7 μm)	_	Calu-3 cell line	_	(Gaspar et al., 2019)
Doxorubicin and miR-519c	PLGA-1.5A (50:50)	W_1_/O/W_2_ emulsification	42.7 − 47.6 μm	cumulative release values of doxorubicin and miR-519c were 80% and 50% respectively after 7 days	A549 cells	_	(Wu et al., 2016)
Budesonide	RG503H (PLGA 50:50, Mw: 28KDa)	Premix membrane O/W emulsification	3 μm	7 days	RAW264.7 cells and NR8383 cells	Male Sprague Dawley rats	(Li et al., 2019)
Gatifloxacin	PLGA 50:50 (RG502 and RG502H, Mw: 12KDa)	O/W emulsification	3–5 μm	cumulative release of 73–75% after 2 days	raw 264.7 mouse macrophage cell line	_	(Marcianes et al., 2019)

### 4.4. PLGA microsphere in ocular drug delivery

The obstacles to the delivery of ocular drugs mainly come from the static (corneal layers, sclera, retina, blood-aqueous and blood-retinal barriers) and dynamic barriers (tear dilution, conjunctival and choroidal blood flow, and lymphatic clearance) of the eye, which limit the distribution of active substances (Patel et al., 2013; Bravo-Osuna et al., 2018; Huang et al., 2020). It is difficult to maintain effective drug concentration continuously during treatment. Systemic administration, eye drops, or injection administration methods not only has low bioavailability, but repeated administration will have systemic side effects and easily cause ocular complications such as endophthalmitis, cataracts, and retinal detachment (Jager et al., 2004; Rong et al., 2014). Therefore, it is necessary to develop a continuous drug delivery system with high safety in the treatment of ocular diseases. Xianfang Rong et al. (Rong et al., 2014) injected PLGA microspheres with low, medium, and high doses and PLGA microspheres loaded with erythropoietin into the vitreous of normal New Zealand rabbits. Researchers took out animal retinal tissues at different time points (1, 2, 4, 8, and 12 weeks) for analysis. The results showed that PLGA based biodegradable microspheres and their natural degradation products did not cause retinal cell death, glial cell activation, or inflammatory reaction, nor did they cause histological changes and functional damage of the retina. Therefore, PLGA microspheres have good biocompatibility and safety in the treatment of eye diseases and can be used as a continuous ophthalmic drug delivery system. At present, there have been many studies on PLGA biodegradable microspheres in ocular drug delivery. A. Aarranz-Romera et al. (Arranz-Romera et al., 2019) developed a kind of PLGA microspheres (mean particle size of 29.04 ± 1.89 μm, EE% > 62%) that could simultaneously load three neuroprotective agents (dexamethasone, melatonin, and coenzyme Q10) for the treatment of glaucoma. They reported that the microspheres (DMQ-MS_S_) were able to continuously deliver the encapsulated active compounds in the form of low burst release within 30 days after intravitreal injection. They demonstrated the neuroprotective effect of DMQ-MSs in a glutamate-induced cytotoxicity model in the R28 cell line (IC50 10.00 ± 0.94 mM versus 6.89 ± 0.82 mM in absence of DMQ-MSs). Then, they used a well-established rodent (rat) model of chronic ocular hypertension (OHT) to study the pharmacodynamics in vivo. It was found that DMQ-MSs showed an obvious neuroprotective effect compared with the OHT treated control group, while the blank microspheres group and single drug-loaded microspheres group had no protective effect. In another study, Rajat Chauhan et al. (Chauhan et al., 2019) used spray drying to develop dasatinib-loaded PLGA microspheres for the treatment of proliferative vitreoretinopathy. By optimizing the spray drying process parameters, particles with a particle size of 0.5–1.5 μm were obtained. They reported that the release time for particles with an average particle size > 1.0 μm could be up to 55 days, while the release time for sub-micron particles was 15 days. In-vitro cell contraction test showed that dasatinib-loaded PLGA microspheres could significantly inhibit the contraction to less than 10% regardless of their size. Then, they simulated the environment of the eye fluid and found that the therapeutic effect of dasatinib-loaded PLGA microspheres was better than that of a single dasatinib solution injection. Laura Ferna′ndez SA′nchez et al. (Fernandez-Sanchez et al., 2017) prepared PLGA microspheres (mean particle size of 23 μm) loaded with tauroursodeoxycholic acid (TUDCA). The therapeutic effect was evaluated by intravitreal injection of the microspheres into the rat model of retinitis pigmentosa. They observed scotopic ERG responses to assess the effect of TUDCA-loaded PLGA MSs on the responsiveness of P23H rat retinas by injecting TUDCA-PLGA into the right eye of the rat model without treating the left eye. Compared with the control group, the ERG responses in the eyes injected with TUDCA-PLGA MSs were lighter. TUDCA-PLGA showed preserved synaptic interactions between photoreceptor cells and secondary neurons: bipolar and horizontal cells. In conclusion, the prepared TUDCA-PLGA could alleviate vision loss and retinal remodeling in animal models of retinal degeneration. In addition to using a single PLGA loading material as an ocular drug delivery carrier, some studies use two or more materials combined with PLGA as a drug delivery carrier. Rui Guo et al. (Guo et al., 2018) prepared 3 D PLGA/silica colloidal crystal microparticles, which could be used for drug delivery and drug release monitoring at the same time. The microparticles were loaded with dexamethasone (DEX) as a therapeutic agent for the treatment of proliferative vitreoretinopathy (PVR). The microparticle system was first self-assembled by silica nanoparticles into a three-dimensional porous structure with a large specific surface area. At this time, the microparticles presented a bright structural color, and then PLGA and DEX were deposited in the particle voids. When the deposition was complete, the structural color fades. With the slow release of the drug and the automatic degradation of PLGA, the color of the microparticles would gradually recover, thus completing the drug release monitoring process. In the cytotoxicity test, they used a drug-free (DEX) particle system and found that the cell survival rate was above 95%. In the cell proliferation experiment, the drug-loaded microparticles exhibited a significant advantage in inhibiting the proliferation of ARPE-19 cells due to their sustained release ability. In conclusion, PLGA microspheres show great potential in ocular drug delivery and can be used as a high-safety drug delivery system for the continuous delivery of drugs to the eye (Table 5).

**Table 5.** PLGA-based biodegradable microspheres for ocular drug delivery.

Drugs	PLGA composition	Preparation method	Sustained drug release	Cell model	Animal model	References
Dexamethasone, melatonin, and coenzyme Q10	PLGA 50:50 (M_W_: 35,000 g/mol)	O/W emulsification	30 days	R28 cell line	Adult male Dark Agouti rats	(Arranz-Romera et al., 2019)
Dasatinib	Ratios and weight: PLGA 50:50, PLGA 65:35, and PLGA 75:25 (M_W_: 30–60, 50–80, and 65–120 KDa, respectively.) Concentration: 0.55–0.65 w/v% (PLGA in DCM)	Spray drying	Particle size >1.0 μm：55 days sub-micron: 15 days	Swine RPE cells	_	(Chauhan et al., 2019)
Tauroursodeoxycholic acid	PLGA 50:50	O/W emulsification	28 days with a cumulative release of 40.5%	_	Homozygous P23H line 3 albino rats	(Fernandez-Sanchez et al., 2017)
Dexamethasone	PLGA 50:50 (M_W_: 5 KDa)	Microfluidic technology	4 weeks with a cumulative release of 94.9%,	ARPE-19 cells	_	(Guo et al., 2018)

## 5. Conclusion

In this review, we have discussed the selection of carrier materials PLGA and introduced the preparation methods of PLGA biodegradable microspheres. More importantly, we summarized their applications in drug delivery. The composition, molecular weight, and concentration of PLGA will affect the size, drug loading capacity, and release curve of PLGA microspheres. Researchers should consider the physicochemical properties of drugs and the characteristics of PLGA when selecting materials. Choosing the right PLGA carrier material is helpful to realize the ideal structure and function of PLGA microspheres. In the future, PLGA polymers with specific applicability or degradation kinetics can be designed according to the specific structure or function of PLGA microspheres. Each preparation method has its advantages and disadvantages. During the preparation of microspheres, suitable methods can be selected according to the nature of the encapsulated drug and the actual operating conditions. The corresponding improvement measures are also summarized because of the shortcomings of some preparation methods. The review focuses on the research of PLGA microspheres in the delivery of cancer drugs, protein or peptide drugs, pulmonary and ocular drug delivery, as well as the application simulation in in-vivo and in-vitro models, showing its great potential in the field of biomedicine. It is also pointed out that the selection of in-vivo and in-vitro animal models that are more in line with the actual disease application simulation requirements will be more conducive to the transformation of research results to clinical trials.

PLGA-based biodegradable microspheres have been widely used in drug delivery in the past decades, and tens of products have been approved for market application. With the improvement of biodegradable microsphere design and efficacy, the scope of application will continue to expand in the future, especially in the delivery of anti-cancer drugs and protein or peptide drugs, and so on. The main advantages of PLGA-based biodegradable microspheres lie in their biocompatibility, stability, and biodegradability. They can be used to encapsulate various types of drugs and deliver the loaded drugs to specific sites in the required way. Their drug release kinetics can also be specifically adjusted by some methods. However, there are still some technical barriers that are difficult to overcome, such as the difficulty of encapsulation of certain properties drugs and the production of microspheres, and the difficulty of transforming research results to clinical, which are the problems that need to be focused on and solved in the future research stage. With the further development of biodegradable microsphere research and the increasing demand, it is believed that PLGA-based biodegradable microspheres will continue to be a popular drug delivery carrier in the future and will be increasingly used in clinical and practical disease treatment.
